# Feline paraneoplastic hypereosinophilic syndrome associated with lymphocytic intestinal lymphoma

**DOI:** 10.29374/2527-2179.bjvm000926

**Published:** 2026-07-08

**Authors:** Marina Moller Nogueira, Matheus Vilardo Lóes Moreira, Ana Flávia Machado Botelho, Paulo Ricardo Oliveira Paes, Marília Martins Melo

**Affiliations:** 1 Departamento de Clínica e Cirurgia Veterinárias, Escola de Veterinária, Universidade Federal de Minas Gerais (UFMG), Belo Horizonte, MG, Brazil.; 2 Cristalino Centro de Diagnóstico Veterinário, Belo Horizonte, MG, Brazil.; 3 Departamento de Clínica e Cirurgia Veterinárias, Escola de Veterinária e Zootecnia, Universidade Federal de Goiás (UFG), Goiânia, GO, Brazil.

**Keywords:** cat, lymphocytic alimentary lymphoma, hypereosinophilia, gato, linfoma alimentar linfocítico, hipereosinofilia

## Abstract

Alimentary lymphocytic lymphoma (AL) is the most prevalent neoplastic subtype in cats. T-cell neoplasms have been associated with eosinophil chemotaxis and, consequently, paraneoplastic hypereosinophilic syndrome. This report describes a case of marked hypereosinophilia in a cat with AL, consistent with a paraneoplastic hypereosinophilic syndrome, based on clinical findings. A 13-year-old neutered male Persian cat, negative for feline immunodeficiency virus (FIV) and feline leukemia virus (FeLV), with a history of frequent vomiting, presented with a mild increase in abdominal volume on palpation, suggestive of mesenteric lymph node enlargement. Blood profile revealed marked leukocytosis (81,500 leukocytes/µL) due to severe eosinophilia (69,000/µL), consisting exclusively of segmented eosinophils. Abdominal ultrasonography showed lymphadenomegaly and increased echogenicity of the meso-omentum, suggestive of an infiltrative neoplastic process. Following suspicion of lymphoma, intestinal biopsy and bone marrow (BM) sampling were performed. BM evaluation was consistent with a myeloproliferative process, with eosinophilic lineage involvement. Histopathological examination of the duodenum, jejunum, and mesenteric lymph node confirmed the diagnosis of AL. Treatment with prednisolone, chlorambucil, and cobalamin was instituted. One month after treatment, the cat was clinically stable, with a total eosinophil count of 22,000/µL. After six months, eosinophil counts decreased to 9,030/µL, and abdominal ultrasonography showed no abnormalities. This report highlights the importance of investigating AL in cats presenting with marked hypereosinophilia.

## Resumo

O linfoma linfocítico alimentar (AL) é o subtipo neoplásico mais prevalente em gatos. Neoplasias de células T têm sido associadas à quimiotaxia de eosinófilos e, consequentemente, à síndrome hipereosinofílica paraneoplásica. Este relato descreve um caso de hipereosinofilia acentuada em um gato com AL, compatível com síndrome hipereosinofílica paraneoplásica, com base em achados clínicos. Um gato Persa, macho, castrado, de 13 anos de idade, negativo para o vírus da imunodeficiência felina (FIV) e o vírus da leucemia felina (FeLV), com histórico de vômitos frequentes, apresentou leve aumento do volume abdominal à palpação, sugestivo de aumento de linfonodos mesentéricos. O hemograma revelou leucocitose acentuada (81.500 leucócitos/µL) decorrente de eosinofilia severa (69.000/µL), composta exclusivamente por eosinófilos segmentados. A ultrassonografia abdominal evidenciou linfadenomegalia e aumento da ecogenicidade do meso-omento, sugestivos de processo neoplásico infiltrativo. Diante da suspeita de linfoma, foram realizadas biópsias intestinais e coleta de medula óssea (MO). A avaliação da MO foi compatível com processo mieloproliferativo, com envolvimento da linhagem eosinofílica. O exame histopatológico do duodeno, jejuno e linfonodo mesentérico confirmou o diagnóstico de AL. O tratamento com prednisolona, clorambucil e cobalamina foi instituído. Após um mês, o animal encontrava-se clinicamente estável, com contagem total de eosinófilos de 22.000/µL. Após seis meses, a contagem de eosinófilos reduziu para 9.030/µL, e a ultrassonografia abdominal não evidenciou alterações. Este relato destaca a importância de investigar AL em gatos com hipereosinofilia acentuada.

## Introduction

Lymphoma is the most common hematopoietic neoplasm in veterinary medicine, accounting for approximately 80% of malignant tumors diagnosed in domestic animals (Valli et al., 2000; Barrs & Beatty, 2012). In cats, lymphoma represents nearly one-third of all malignant neoplasms, with the alimentary form being the most frequently diagnosed (Norsworthy, 2018).

Alimentary lymphoma (AL) may present as a solitary, multifocal, or diffuse infiltrative process affecting the gastrointestinal tract and may or may not involve mesenteric lymph nodes. The most common clinical signs include vomiting, weight loss, and diarrhea (Gieger, 2011). Three histological grades are recognized: low-grade (small-cell, lymphocytic), intermediate-grade, and high-grade (large-cell, lymphoblastic) lymphoma. Large granular lymphocyte lymphoma is considered a subclassification of AL and may present at any grade (Barrs & Beatty, 2012).

Cats with lymphocytic AL, the most common subtype, accounting for approximately 77% of cases (Wolfesberger et al., 2018), generally respond well to chemotherapy, with reported median survival times ranging from 330 to 704 days (Kiselow et al., 2008; Stein et al., 2010).

Hypereosinophilic syndromes are uncommon in cats and may be classified as idiopathic, neoplastic, or paraneoplastic (Barrs et al., 2002). Paraneoplastic hypereosinophilia most commonly occurs secondary to mast cell tumors and lymphomas.

The aim of this study is to report a case of lymphocytic intestinal lymphoma associated with paraneoplastic hypereosinophilic syndrome in a cat and to describe the clinicopathological features of both conditions.

## Case description

A 13-year-old neutered male Persian cat, negative for FIV and FeLV, was presented with a four-month history of frequent vomiting. On physical examination, the cat was alert, with a body condition score of 4/5, capillary refill time <2 seconds, normal mucous membranes, unremarkable oral cavity, normal cardiac and pulmonary auscultation, and no enlargement of peripheral lymph nodes. Abdominal palpation revealed a mild increase in volume, suggestive of mesenteric lymphadenomegaly. The cat had been dewormed two months prior and showed no clinical signs suggestive of allergic disease.

Hematological evaluation revealed marked leukocytosis due to eosinophilia (40,800 leukocytes/µL, with 28,152 eosinophils/µL; reference range: 0-1,500 eosinophils/µL; Feldman et al., 2000). On repeat examination, leukocyte counts doubled to 81,500 leukocytes/µL, with a marked increase in absolute eosinophil count (69,000/µL). All eosinophils were segmented, with no immature forms observed.

Serum biochemical analysis, including urea, creatinine, liver enzymes, glucose, total T4, total protein, albumin, and globulins, revealed values within reference ranges for cats (Thrall et al., 2022).

Abdominal ultrasonography revealed normal thickness and preserved layering of the duodenum, jejunum, and ileum, although increased prominence of the muscularis and submucosal layers suggested an inflammatory or neoplastic process. Multiple enlarged mesenteric lymph nodes with increased meso-omental echogenicity were observed, consistent with an infiltrative process. Given suspicion of lymphoma, thoracic radiographs were obtained for staging and revealed no abnormalities. An exploratory laparotomy was performed for intestinal and lymph node biopsy, as well as bone marrow sampling.

**Figure 1:**
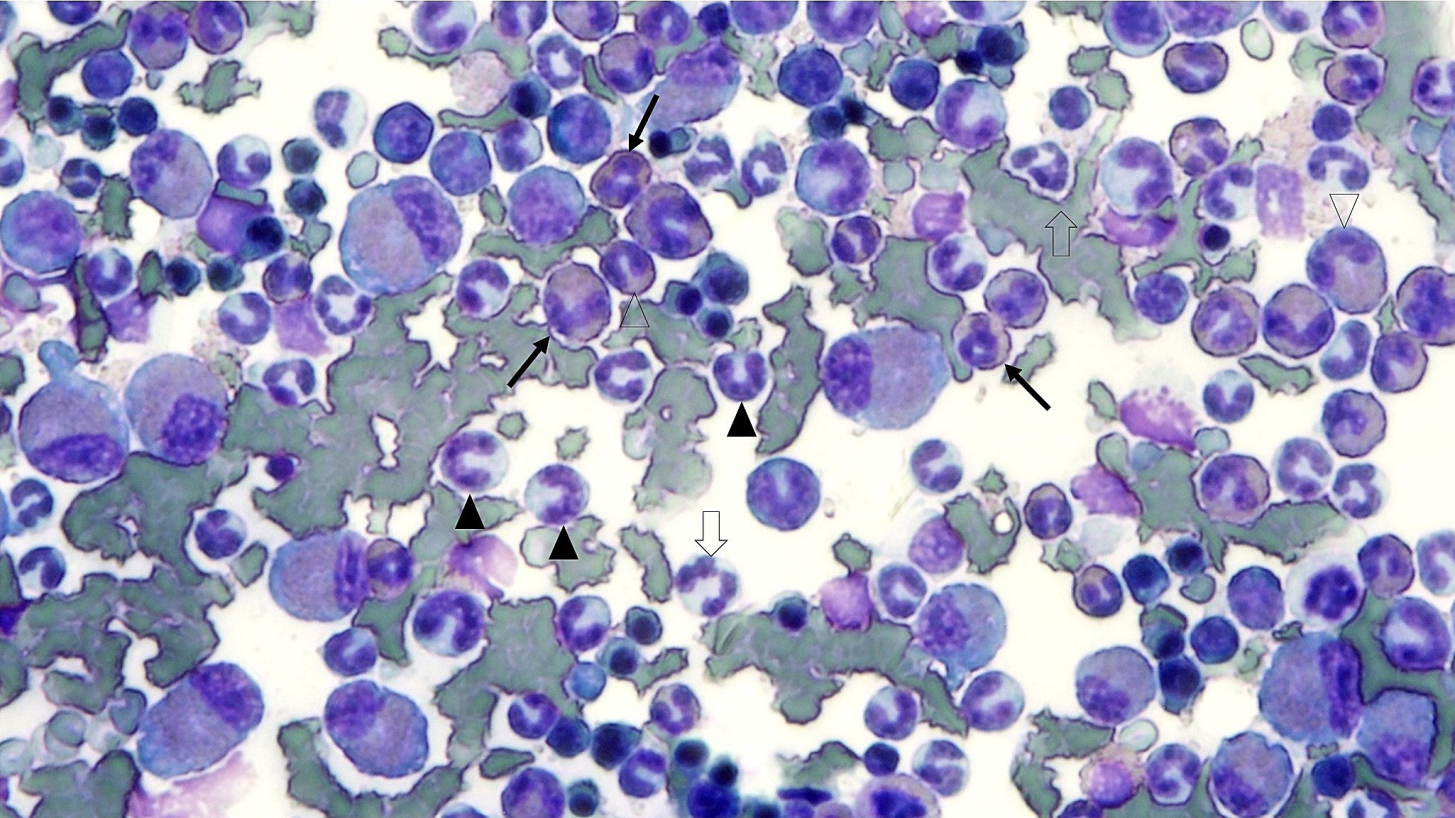
Bone marrow aspirate smear, 40x magnification. The marrow is hypercellular, with an increased myeloid-to-erythroid (M:E) ratio of approximately 5:1. Numerous myeloid precursors are present, including myelocytes, metamyelocytes, and band neutrophils (arrowhead). The eosinophilic lineage is also expanded, with multiple eosinophilic metamyelocytes and band forms (empty arrowhead). Mature segmented eosinophils (arrows) and segmented neutrophils (empty arrows) are relatively decreased, indicating a reduced storage (reserve) pool.

The myelogram revealed a moderately increased overall cellularity, with a cell-to-fat ratio estimated at 50-60% (Figure 1), which is markedly higher than the reference value reported for adult felines (approximately 25.75%) (Cristo et al., 2023). Megakaryocytic density was within the lower limits of the reference range, and no appreciable increase in marrow iron stores was observed upon cytochemical evaluation. Differential assessment of hematopoietic lineages demonstrated a markedly elevated myeloid-to-erythroid (M:E) ratio exceeding 5:1 (reference interval for felines: 0.9-2.2) (Cristo et al., 2023), indicating myeloid predominance. Within the myeloid compartment, the neutrophilic and eosinophilic lineages were proportionally expanded, both exhibiting orderly maturation with a preserved pyramidal distribution of precursor cells. However, a relative reduction in segmented neutrophils and mature eosinophils was noted, suggestive of depletion of the storage (reserve) pool.

Collectively, these findings are indicative of a myeloproliferative pattern, with particular emphasis on eosinophilic lineage expansion. This profile is consistent with reactive (secondary) eosinophilic hyperplasia; however, a primary clonal disorder, such as chronic eosinophilic leukemia, cannot be excluded based solely on cytomorphological criteria. Therefore, definitive differentiation between reactive and neoplastic processes requires integration with clinical findings, peripheral blood evaluation, and, when available, additional diagnostic modalities.

**Figure 2:**
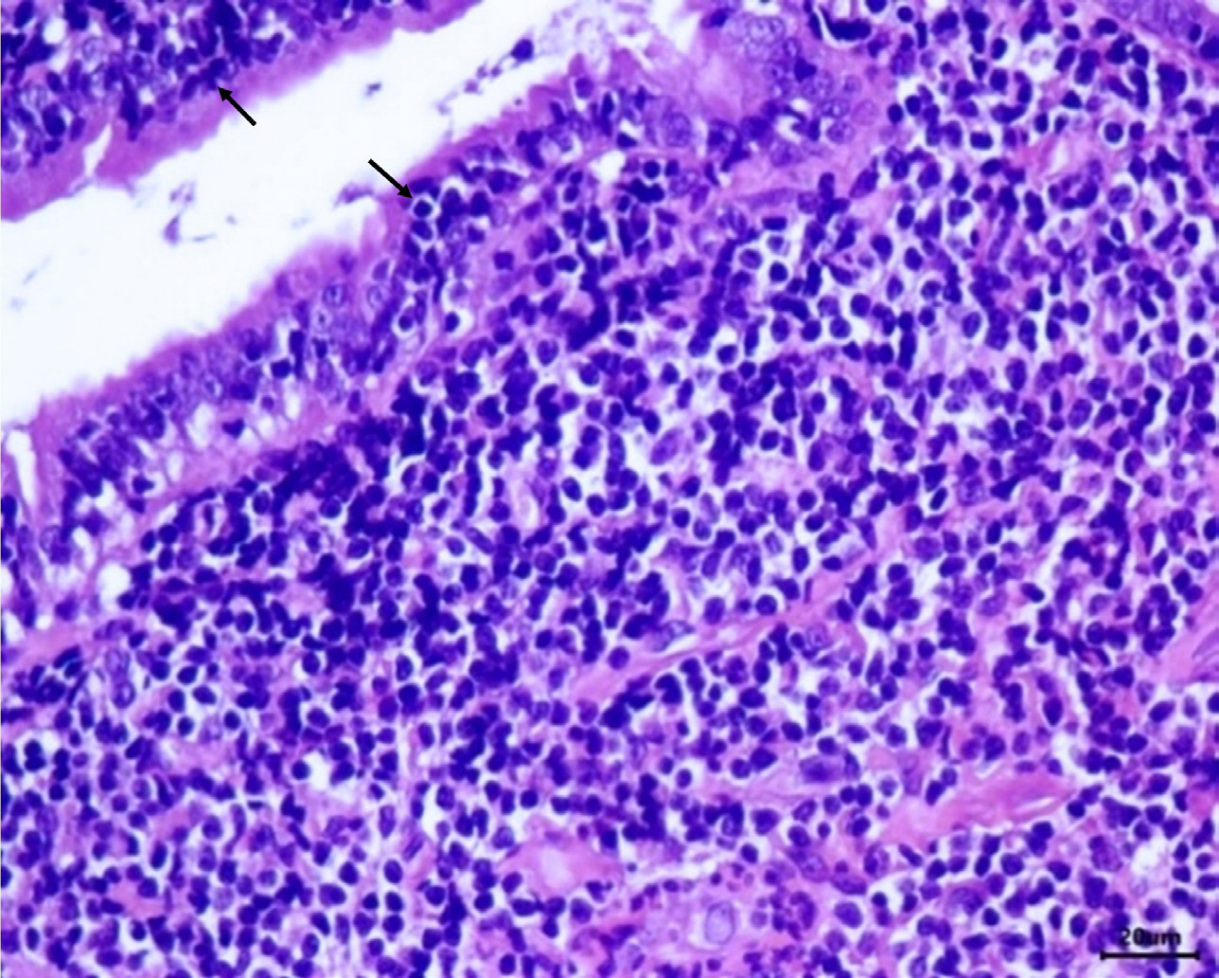
Duodenal tissue section. (A) Low magnification (100x). (B) High magnification (400x). The mucosa exhibits marked cellularity, with dense infiltration by neoplastic lymphoid cells forming cohesive aggregates and nests (arrows), resulting in disruption of the normal tissue architecture.

After the results, it was decided to perform a biopsy of the duodenum, jejunum, and mesenteric lymph node, in which the histopathological examination confirmed the diagnosis of feline lymphocytic alimentary lymphoma. Figure 2 shows photomicrographs of the small intestine, with an intense increase in cellularity in the lamina propria of the villi and in the submucosa, composed of a monotonous proliferation of round neoplastic cells compatible with small lymphocytes. Groupings of neoplastic cells within the villous epithelium were also present, forming nests and plaques (epitheliotropism).

**Figure 3:**
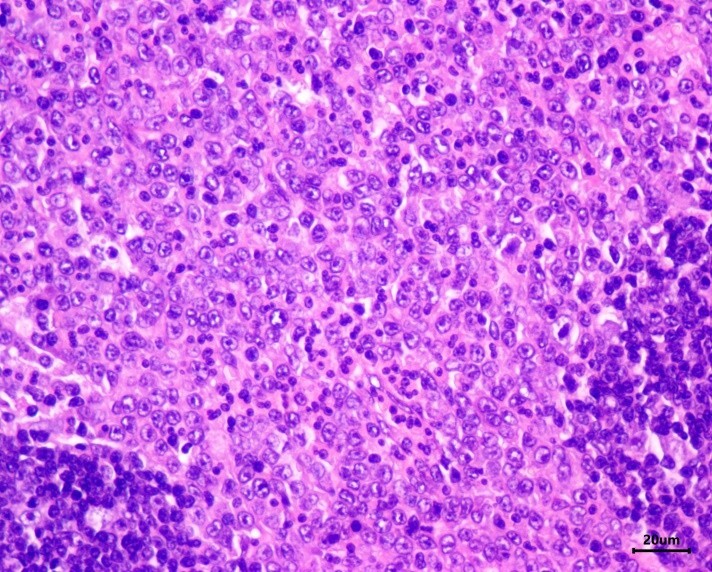
Mesenteric lymph node (extranodal involvement), 400x magnification. There is complete effacement of the normal nodal architecture by a dense population of neoplastic lymphoid cells. The infiltrate extends into the surrounding adipose tissue, which is largely replaced by neoplastic cells.

The mesenteric lymph node (Figure 3) presented loss of tissue architecture, making it impossible to visualize the capsule and sinuses due to infiltration of neoplastic lymphocytes. Infiltration of the neoplasm was observed in the extranodal adipose tissue, where it contained a large quantity of eosinophils and mast cells. Therefore, the mesenteric lymph node was also affected by the neoplasm.

Given the clinical picture and the test results, a therapeutic protocol was instituted based on prednisolone (1 mg/kg every 24 hours) and chlorambucil (2 mg/cat every 48 hours), in addition to weekly application of cobalamin for four weeks, with subsequent monthly application.

One month after treatment initiation, the cat was clinically stable, with eosinophils reduced to 22,000/µL (reference value 0-1.500 eosinophils/µL, according Feldman et al., 2000). After six months, eosinophil counts further decreased to 9,030/µL, and the cat remained clinically stable with unremarkable laboratory and ultrasonographic findings.

## Discussion

Hypereosinophilia refers to a marked increase in eosinophils in the peripheral blood, far above normal values, and may be caused by several conditions in cats. The most common causes include allergies (including flea allergy, food allergy, and asthma), which induce IgE-mediated eosinophilic responses (Balan et al., 2017); parasitic infections (helminths and other parasites that stimulate eosinophilia) (Hotta et al., 2024); and inflammatory or immune-mediated diseases, such as the feline eosinophilic granuloma complex (Taglinger et al., 2005) which do not exceed 50,000/µL (Center et al., 1990). However, neoplastic causes also exist, consisting of tumors that secrete factors that stimulate eosinophils, including lymphoma (especially T-cell lymphoma), mast cell tumors, carcinomas, and other malignant neoplasms. These tumors may trigger hypereosinophilia as a paraneoplastic syndrome.

In the present case, hypereosinophilia was attributed to lymphocytic LA, given that alternative etiologies, including allergic and parasitic disorders, were excluded. Chronic eosinophilic leukemias are exceedingly rare in felines, and a marked reduction in eosinophil counts following the initiation of antineoplastic therapy supports the diagnosis of paraneoplastic hypereosinophilia. Comprehensive clinicopathological evaluation, including a complete blood count and serum biochemical analysis (encompassing hepatic, renal, and thyroid function parameters), is warranted, as concurrent comorbidities or lymphomatous infiltration of these organs may be present. Notably, hypoalbuminemia has been reported in approximately 49% of cats with lymphocytic LA, likely secondary to intestinal protein loss, resulting in hypoproteinemia (Gieger, 2011); however, this alteration was not observed in the present case.

Paraneoplastic hypereosinophilia is an abnormal hematologic response secondary to the presence of a tumor, without eosinophils being part of the neoplasm itself. Instead, the neoplasm secretes molecules that stimulate the production and activation of eosinophils in the bone marrow and tissues. Case reports have described hypereosinophilia associated with intestinal T-cell lymphoma in cats, frequently accompanied by eosinophilic infiltration of affected tissues. Barrs et al. (2002) reported hypereosinophilic syndrome associated with intestinal T-cell lymphoma, characterized by marked peripheral blood eosinophilia and the presence of eosinophil accumulations in the mesentery, lymph nodes, and intestine. Cave et al. (2004) described a case of epitheliotropic lymphoma with paraneoplastic eosinophilia, reinforcing the clinical association of this hematologic response with feline lymphoma. In addition, Balan et al. (2017) reported a cat with alimentary T-cell lymphoma that exhibited peripheral eosinophilia with concurrent basophilia, indicating that these responses may coexist.

Although the exact pathophysiology in cats remains poorly understood, the proposed mechanisms are extrapolated from feline, canine, and human cases. Tumor-derived cytokine stimulation may occur, particularly involving interleukin-5 (IL-5), a key cytokine in eosinophil differentiation, growth, and survival. Tumor cells, especially neoplastic T lymphocytes, may secrete IL-5, thereby promoting systemic eosinophilia (Barrs et al., 2002). Other cytokines, such as IL-3 and granulocyte-macrophage colony-stimulating factor (GM-CSF), may also contribute to eosinophil proliferation and chemotaxis, although these have been less specifically documented in cats (Balan et al., 2017). Tissue recruitment of eosinophils may also occur, as tumors can secrete chemotactic factors that attract eosinophils to neoplastic or inflammatory tissues, resulting in eosinophilic infiltrates in the tumor microenvironment and in lymph nodes (Barrs et al., 2002).

Similarities have been described in humans and other species. In people with T-cell lymphoma and other neoplasms, hypereosinophilia has been associated with abnormal cytokine production by malignant cells, indicating a shared pathogenic mechanism (McNaught et al., 2018). In dogs and cats, although rare, reported cases support the concept that dysregulation of normal eosinophil control by tumors can result in this syndrome (McNaught et al., 2018).

Lymphoma is the most common neoplasm in cats, with alimentary lymphoma (AL) representing the predominant anatomical form and the lymphocytic subtype being the most prevalent (Wilson, 2008). Gastrointestinal involvement accounts for approximately 31% of all feline lymphomas (Cesari et al., 2009), which is consistent with the findings in this case, including mild abdominal enlargement on palpation and mesenteric lymphadenomegaly detected on ultrasonography. Mesenteric lymph node enlargement is reported in 47-75% of cats with AL (Lingard et al., 2009; Zwingenberger et al., 2010). Although intestinal wall thickening is described in up to 81% of affected cats (Lingard et al., 2009), this alteration was not observed in the present case, highlighting the variability of ultrasonographic findings.

Cats with lymphocytic AL range in age from 2 to 20 years, with a higher prevalence between 10 and 13 years (Barrs & Beatty, 2012; Freiche et al., 2021), consistent with the age of the cat described herein. Male cats and Siamese or Oriental breeds have been reported to have an increased predisposition to lymphoma (Stein et al., 2010; Rissetto et al., 2011), supporting the observed gender susceptibility in this case.

Clinically, lymphocytic AL is characterized by chronic, slowly progressive, and nonspecific signs, most commonly vomiting, anorexia, weight loss, and diarrhea (Kiselow et al., 2008; Gieger, 2011; Lingard et al., 2009; Pope et al., 2015). The four-month history of frequent vomiting in this cat is consistent with these reports. Diagnosis is often challenging and requires integration of clinical findings, laboratory data, imaging, histopathology, and, when available, immunophenotyping. Lymphocytic AL and inflammatory bowel disease (IBD) share overlapping clinical and imaging features, with both conditions frequently presenting with signs lasting several months (Paulin et al., 2018). Additionally, feline lymphomas often arise in tissues affected by chronic inflammation, suggesting a possible pathogenic association (Louwerens et al., 2005).

Lymphocytic AL can involve any segment of the gastrointestinal tract, and more than one site is affected in approximately 94% of cases, particularly the ileum, duodenum, and mesenteric lymph nodes (Lingard et al., 2009). Similarly, Wolfesberger et al. (2018) reported small intestinal involvement in 91% of cats with alimentary T-cell lymphoma, with mesenteric lymph node enlargement in 19% of cases, findings comparable to those observed in this report.

Small- to intermediate-cell T-cell lymphoma (WHO EATCL type II) represents the dominant form of gastrointestinal lymphoma in cats (Kiupel et al., 2011; Moore et al., 2012). T-cell neoplasms may promote eosinophil chemotaxis through cytokine production, resulting in paraneoplastic hypereosinophilic syndrome (Barrs et al., 2002). In the present case, marked peripheral eosinophilia and bone marrow eosinophilic hyperplasia were identified. While eosinophilia is relatively common in cats and may be associated with parasitic, allergic, infectious, immune-mediated, or neoplastic diseases, hypereosinophilic syndrome is rare and is defined by persistent peripheral and tissue eosinophilia with associated organ involvement (Barrs et al., 2002).

Hypereosinophilic syndrome encompasses several variants, including myeloproliferative, lymphocytic, genetic, and secondary forms, and includes chronic eosinophilic leukemia as a subtype. The lymphocytic variant is characterized by reactive eosinophilia secondary to abnormal T-lymphocyte proliferation and cytokine secretion, particularly IL-3, IL-5, and GM-CSF (Takeuchi et al., 2012). Importantly, differentiation between chronic eosinophilic leukemia and paraneoplastic hypereosinophilia cannot be achieved based solely on myelogram findings and requires clinical correlation.

Intestinal lymphocytic lymphomas may be associated with moderate to severe eosinophilia and eosinophilic infiltration of the intestinal wall and mesenteric lymph nodes (Barrs & Beatty, 2012). Although paraneoplastic eosinophilia is most frequently described in association with mast cell tumors, it has also been reported in lymphomas and carcinomas (Balan et al., 2017). In cats, non-neoplastic eosinophilia related to allergic or inflammatory conditions typically does not exceed 50,000/µL (Center et al., 1990), whereas extreme eosinophilia may occur in rare conditions such as paraneoplastic hypereosinophilic syndrome or eosinophilic leukemia (Gelain et al., 2006). In this case, alternative causes of eosinophilia were excluded, and the marked reduction in eosinophil counts following lymphoma treatment strongly supports a paraneoplastic origin.

Abdominal ultrasonography remains the imaging modality of choice for evaluation of intestinal disease in cats. Although ultrasonographic abnormalities are present in over 90% of cats with lymphocytic AL, differentiation from IBD is not possible based on imaging alone (Moore et al., 2012). Mesenteric lymphadenomegaly, observed in up to 75% of cats with AL but only 17% of cats with IBD, may provide additional diagnostic support (Lingard et al., 2009; Zwingenberger et al., 2010; Gieger, 2011).

Definitive diagnosis of lymphocytic AL requires histopathological evaluation of intestinal biopsies, which may be obtained via laparotomy or endoscopy. Laparotomy allows full-thickness sampling and assessment of extraintestinal tissues, including mesenteric lymph nodes, liver, and pancreas, whereas endoscopic biopsy is less invasive but limited to mucosal layers (Gieger, 2011; Moore et al., 2012). Histologically, lymphocytic AL is characterized by infiltration of neoplastic lymphocytes within the intestinal mucosa, often with progression to deeper layers and epitheliotropism, as observed in this case (Richter, 2003; Gieger, 2011).

Treatment with chlorambucil and prednisolone is considered first-line therapy for lymphocytic AL and is associated with favorable outcomes (Wilson, 2008). Reported median survival times range from 428 to 897 days, with remission achieved in up to 96% of cases (Kiselow et al., 2008; Stein et al., 2010). In the present case, the therapeutic protocol resulted in clinical remission, normalization of imaging findings, and a marked decrease in eosinophil counts, indicating a favorable prognosis.

This case underscores the diagnostic challenge posed by severe eosinophilia and highlights the importance of considering alimentary lymphoma as an underlying cause of hypereosinophilia in cats.

## Conclusions

Lymphocytic alimentary lymphoma is a common neoplasm in older cats and often presents with chronic, nonspecific clinical signs. Its diagnosis is challenging due to overlap with inflammatory bowel disease. The findings in this case support a diagnosis of paraneoplastic hypereosinophilia secondary to lymphocytic alimentary lymphoma. Successful treatment of the neoplasm resulted in marked improvement of hypereosinophilia. This report emphasizes the importance of investigating alimentary lymphoma in cats presenting with severe eosinophilia.
